# Antibiotic alternatives: the substitution of antibiotics in animal husbandry?

**DOI:** 10.3389/fmicb.2014.00217

**Published:** 2014-05-13

**Authors:** Guyue Cheng, Haihong Hao, Shuyu Xie, Xu Wang, Menghong Dai, Lingli Huang, Zonghui Yuan

**Affiliations:** ^1^MOA Laboratory for Risk Assessment of Quality and Safety of Livestock and Poultry Products, Huazhong Agricultural UniversityWuhan, China; ^2^National Reference Laboratory of Veterinary Drug Residues, Huazhong Agricultural UniversityWuhan, China; ^3^MOA Key Laboratory for the Detection of Veterinary Drug Residues in Foods, Huazhong Agricultural UniversityWuhan, China

**Keywords:** antibiotics, antibiotic alternatives, application, limitation

## Abstract

It is a common practice for decades to use of sub-therapeutic dose of antibiotics in food-animal feeds to prevent animals from diseases and to improve production performance in modern animal husbandry. In the meantime, concerns over the increasing emergence of antibiotic-resistant bacteria due to the unreasonable use of antibiotics and an appearance of less novelty antibiotics have prompted efforts to develop so-called alternatives to antibiotics. Whether or not the alternatives could really replace antibiotics remains a controversial issue. This review summarizes recent development and perspectives of alternatives to antibiotics. The mechanism of actions, applications, and prospectives of the alternatives such as immunity modulating agents, bacteriophages and their lysins, antimicrobial peptides, pro-, pre-, and synbiotics, plant extracts, inhibitors targeting pathogenicity (bacterial quorum sensing, biofilm, and virulence), and feeding enzymes are thoroughly discussed. Lastly, the feasibility of alternatives to antibiotics is deeply analyzed. It is hard to conclude that the alternatives might substitute antibiotics in veterinary medicine in the foreseeable future. At the present time, prudent use of antibiotics and the establishment of scientific monitoring systems are the best and fastest way to limit the adverse effects of the abuse of antibiotics and to ensure the safety of animal-derived food and environment.

## INTRODUCTION

Since the discovery and application of penicillin in 1940s, antibiotics have played unparalleled roles in the prevention, control, and treatment of infectious diseases for humans and animals. It is also proved that the use of antibiotics in animal feeds is an important way to enhance feed efficiency, to promote animal growth, and to improve the quality of the animal products. Recent studies showed that the growth-promoting effect of antibiotics was correlated with the decreased activity of bile salt hydrolase, an intestinal bacteria-produced enzyme that exerts negative impact on host fat digestion and utilization (Lin, 2014). Therefore, antibiotics are effective tools for ensuring the development of intensive and large-scale farming industry. However, the unreasonable use of antibiotics has given rise to a fear of the development of resistant bacteria (Aarestrup et al., 1998) that may lead to the transfer of resistant bacteria and its resistant factors from animals to humans (Stanton, 2013). Non-therapeutic antimicrobial uses are also linked to the propagation of multidrug resistance (MDR), including resistance against drugs that were never used on the farm (Marshall and Levy, 2011). Due to this concern, Sweden firstly prohibited the use of some of the antibiotics in animal feeds in 1986 (Castanon, 2007), and European Union (EU) member nations banned all antibiotic growth promoters in 2006 according to European Parliament and Council Regulation EC No. 1831/2003.

However, the ban of in-feed use of antibiotics has brought unintended impacts on animal production industries in EU, such as the increase of infections in animals and the decrease of animal production. Meanwhile, the total usage amount of antibiotics in animals increased because the use of therapeutic antibiotics and disinfectants was significantly increased due to the fact that the high incidence of diseases occurred resulted from the ban. Unlike its golden age when a lots of antibiotics were discovered and commercialized, the discovery and development of new antibiotics dramatically decreased for decades (Stanton, 2013). The antimicrobial shortages increased by 283% in 2006~2010 (Borchardt and Rolston, 2013). The lack of novel core moiety of antibiotics potentially compensate for the resistance to existing antibiotics and owes to the high cost and risk associated with the development and application of such products (Cooper and Shlaes, 2011).

To overcome the increased rate of mortality and morbidity due to the ban of in-feed antibiotics, a number of alternatives/replacements have been proposed (Seal et al., 2013). They are antibacterial vaccines, immunomodulatory agents, bacteriophages and their lysins, antimicrobial peptides (AMPs), pro-, pre-, and synbiotics, plant extracts, inhibitors for bacterial quorum sensing (QS), biofilm and virulence, and feed enzymes, etc. (Millet and Maertens, 2011). Are these antibiotic alternatives really as effective as antibiotics to control the diseases in animals? The development and application of the alternatives to antibiotic was reviewed, and the possibility of the alternatives to antibiotics was discussed in this paper.

## IMMUNITY MODULATING AGENTS

The development of an infection is the interaction between the pathogen and the immune system of the host. The immune system protects the body against the disease by recognizing and neutralizing the pathogen. The innate immune response includes both humoral and cellular defense such as the complement system and the processes played by granulocytes and macrophages. Immunity modulating agents (immunomodulators) are used for immunotherapy, which is defined as treatment of disease by inducing, enhancing, or suppressing an immune response. Vaccine is one of the most important immunomodulators, and some pharmaceutical agents could also be used as immunomodulators.

### ANTIBACTERIAL VACCINES

Traditional vaccines are generally classified into live-attenuated and inactivated/killed vaccines. Bacterin is a suspension of killed or weakened bacteria used as a vaccine. Live-attenuated bacteria, replicating transiently in the host, are capable of expressing a full repertoire of antigens. Take *Salmonella* vaccine for an example. Many live *Salmonella* vaccine strains have been tested with varying degrees of efficacies (Desin et al., 2013). However, the major drawbacks of the live strains is that they persist in the animal body for a longer time (Tan et al., 1997) and have a high risk of reverting to full virulence (Barrow, 2007; Gast, 2007). Although various *Salmonella* live-attenuated vaccines have been reported, not all of them have been tested under field conditions. In addition, they do not induce sufficient cross protection against other non-host-adapted serotypes (Desin et al., 2013).

Killed vaccines are safer than the live vaccines. It is made by killing *in vitro*-grown bacterial cultures and packing with oil-based adjuvants to enhance immune responses (Potter et al., 2008). They are quite inexpensive in production and stable in storage. The inactivated autogenous poultry vaccines currently include *Pasteurella multocida*, *Salmonella*, *Mycoplasma*, *Ornithobacterium*, *Haemophilus*, *Staphylococcus*, *Escherichia coli*, and *Bordatella Bacterins*. However, killed vaccines have numerous disadvantages such as the lack of relevant protective antigens (PAs) due to *in vitro* growth conditions and killing processes (Barrow, 2007; Gast, 2007), antigenic competition between non-protective and protective components, a lack of safety due to potentially harmful components such as lipopolysaccharide, and a lack of broad-spectrum protection. In addition, killed vaccines require the use of adjuvants which limits the delivery options for the vaccines. Moreover, most of the killed vaccines are injectable products and are not routinely used in intensive broiler operations.

With the increasing use of bacterins, there are concerns that this may lead to the increasing virulence of bacteria. As an alternative, subunit vaccine is composed of either a single antigen or multiple defined antigens (predominantly proteins). This kind of vaccines lack the regulatory and biological complications associated with the living organisms. On the other hand, subunit vaccines are usually poorly immunogenic, requiring formulation with appropriate adjuvant(s) (Mutwiri et al., 2011). Although *Salmonella* subunit vaccines are under development, it is hard to conclude that one class of vaccines is more efficacious than another (Desin et al., 2013). Besides, the use of oral subunit vaccines in large animals remains problematic due to the degradation of the antigens and poor absorption in guts (Potter et al., 2008).

DNA vaccines offer another promising improvement to conventional vaccines (Haygreen et al., 2005). DNA vaccine is made up of a small, circular piece of bacterial DNA (called a plasmid) that has been genetically engineered to include the DNA sequence(s) encoding the antigen(s) from a pathogen. When the vaccine DNA is injected into the cells of the body, the host cells “reads” the DNA and converts it into pathogenic proteins which would trigger a range of immune responses. Nevertheless, DNA vaccine is limited in its protective capacity to the encoded proteins on the vector and may pose a risk of integrating the genetic elements of the vector into the host genome. Most of the literatures dealing with DNA vaccines have described the use of viral antigens delivered in mouse models. However, when they are used in large animals, results are often disappointing (Potter et al., 2008). Therefore, DNA vaccines are unlikely to reach the market until the plasmid dose can be controlled and the problems of effective delivery are solved.

Unlike the anti-viral vaccine market which is quite mature, the available antibacterial vaccines are still rare in market. For example, no vaccines are commercially available for either *C. jejuni* or *E. coli* O157 although immunization against both of these strains has been respectively demonstrated in chicken and cattle (Potter et al., 2008). In the case of *E. coli* O157, immunization with bacterins has not shown any protection effect. Although vaccines against *E. coli* and *Brachyspira hyodysenteriae* for treating swine dysentery have been reported (Francis and Willgohs, 1991; Song et al., 2009), safety and efficacy data are still lacking, hindering the commercialization process of these biological products (Ruan et al., 2011). *S. aureus* vaccines are developed for bovine mastitis, but a systematic review evaluating 24 *in vivo* studies suggests that the methodological differences and a lack of more rigid scientific criteria (such as double blind protocols) in some cases hinder the assessment of the efficacy of these vaccines (Pereira et al., 2011). Moreover, the protection rates of animals by antibacterial vaccine in the market are low (Buckley et al., 2010; Crouch et al., 2010).

Last but not least, the development of a vaccine that is both practical and inexpensive so that it can be affordable for use in poor countries is still a key problem (Zhang and Sack, 2012). As for poultry vaccines, the most important challenge for mass immunization is the cost of vaccine as well as the ability in most cases. While vaccines may lessen our reliance on the use of antibiotics, they are complementary rather than a replacement.

### OTHER IMMUNOMODULATORS

Immunomodulators, mainly immunostimulants, are able to non-specifically enhance the innate immune function and to improve the host’s resistance to diseases. The use of immunotherapy in infectious diseases may resulting in modulating the immune response to a microbe (e.g., by using cytokines and cytokine inhibitors), modifying a specific antigen-based response (e.g., using interferons) and minimizing end-organ damage using non-specific anti-inflammatory agents (e.g., steroids; Masihi, 2000). β-Glucans, bacterial products, and plant constituents could directly initiate activation of innate defense mechanisms acting on receptors and triggering intracellular gene(s) that may result in the production of antimicrobial molecules.

There is a variety of immunostimulants, no less than a dozen categories with hundreds of varieties (Table 1). Since 1990s, nucleotides, thymosin, and oregano oil have mainly been used as immunostimulants. Later, probiotics, herbs and their extracts have also become subjects to immunostimulant studies (Thacker, 2010). Studies in animals exhibit significant health benefits by using β-1,3/1,6-glucan (from yeast cell walls) as a feed ingredient to protect animals against microorganisms (Williams et al., 1996). It is suggested that the use of immunostimulants as feed additives can improve the innate defense of animals, providing resistance against pathogens during periods of high stress, such as grading, reproduction, transfer, and vaccination (Bricknell and Dalmo, 2005).

**Table 1:** Classification of immunostimulants.

Category	Variety
Mineral substances	Selenium, zinc, etc.
Vitamins	Vitamin A, vitamin E, vitamin C, etc.
Amino acids	Arginine, leucine, ubenimex, etc.
Chinese herbal medicines	*Astragalus*, *Echinacea*, etc.
Plant polysaccharides	*Astragalus* polysaccharide, lentinan, algal polysaccharides, ganoderan, *Polyporus* polysaccharide, chitosan, etc.
Oligosaccharides	Mannan-oligosaccharides, fructooligosaccharide, etc.
Microbial preparations	BCG vaccine, corynebacterium seedlings, *Lactobacillus*, cholera toxin B subunit, *Mycobacterium phlei*, muroetasin, prodigiosin, etc.
Immunologic adjuvants	Aluminum adjuvant, propolis, liposome, Freund’s adjuvant, etc.
Hormones and hormone-like substances	Growth hormone, thymosin, metallothionein, thymopentin, etc.
Nucleic acid preparations	Polynucleotide, immune ribonucleic acid, etc.
Anthelmintics	Levomisole, metronidazole, etc.
Chemical synthetics	Levomisole, cimetidine, sodium houttuyfonate, imiquimod, pidotimod, ubenimex, tilorone, polyinosinic acid, etc.
Bacterial extracts	β-Glucan, peptidoglycan, lipopolysaccharide, etc.
Biological (cytokines)	Interferon, transfer factor, interleukin, immune globulin, etc.
Others	Bee pollen, bursa extracts, gamma globulin, heat shock protein, poly IC, glycyrrhizin, etc.

Many factors affect the efficacy of immunostimulants. Immunostimulants exhibit different effects in different animal species. They do not reveal a linear relationship between dose and effect, usually more efficient during or prior to infection. Besides the beneficial effects, immunostimulants have so broad effects among which they inhibit the protective aspects of the host immune system (Thacker, 2010). When immunogenic stimulation persists or autoregulatory immune mechanisms cease, adaptive immunologic events can result in immune-mediated processes detrimental to systemic or organ-specific homeostasis (Moore, 2004). It has been proposed in larval fish aquaculture that the delivery of immunostimulants as a feed additive could be of considerable benefit in boosting the animals’ innate defense with little detriment to the developing of fishes. Conversely, immunomodulating a neotenous animal before its immune system is fully formed may adversely affect the development of a normal immune response (Bricknell and Dalmo, 2005). Importantly, most immunomodulators just enhance the immune system of animals, rather than directly kill the bacteria.

Presently, there are no uniform standards for evaluating the efficacy and safety of immunostimulants. It was reported that a reputed immunostimulant composed of *Propionibacterium acnes* extract, *Ochrobactrum intermedium* lipopolysaccharides and Proclin^®^ did not affect the immune system of goats (Morales-delaNuez et al., 2009). The widespread notion of immunostimulatory plant natural products and their potential therapeutic use is rather obscure, suggesting that the product is some sort of “tonic” for the immune system without actually specifying the mechanisms (Gertsch et al., 2011). It is argued that the paradigm of oral plant immunostimulants lacks clinical evidence, originating from primary *in vitro* studies. No conclusive data on orally administered immunostimulants can be found in the scientific literatures up to now. Overall, immunotherapy to modulate the immune response just can be used as an adjunct to the antimicrobial therapy (Kak et al., 2012).

## BACTERIOPHAGES AND THEIR LYSINS

### BACTERIOPHAGES

Bacteriophages are viruses that are parasitic on bacteria, and they have been considered as one of the types of agents to treat bacterial infections for a long time (Wittebole et al., 2014). They were first discovered by Frederick Twort in UK in 1915 and by Félix d’Herell in France in 1917. The first study on the clinical use of phage was published in Belgium in 1921 by Bruynoghe and Maisin who injected staphylococcus-specific phage near the base of the cutaneous boils to treat cutaneous furuncles and carbuncles. The commercial phages was introduced by two companies in the United States and France in 1940s. Recent animal studies show that phage therapy is worth of recognition (O’Flaherty et al., 2009). It is reported that phages has certain preventive effects on pathogens as *E. coli* O157:H7, *Salmonella* and *Campylobacter* (Huff et al., 2005; Johnson et al., 2008). In 2006, a phage cocktail designated LMP-102^TM^ containing six types of pure bacteriophages was approved by US-FDA as a food additives for prevention of meat contamination with *Listeria*. In 2007, United States Department of Agriculture (USDA) approved another phage product to be used for disinfection of *E. coli* in hidden parts of cattle. Nonetheless, most of the bacteriophage products to date are still in the research stage.

Bacteriophages can replicate in host cells and are able to produce new lytic phages to keep pace with the mutation of pathogens. However, the replacement of antibiotics by phages in treatment of bacterial diseases encounters controversy because of the following characteristics (Pirnay et al., 2011):

(1) Since phages have strict host strain specificity, the precise etiological microorganism causing infection needs to be determined with accuracy before the use of phage therapy (Allen et al., 2013). Also, the narrow host range impedes the ability of a single kind of phages to be used as replacement of antibiotics, which are typically broader in their antibacterial spectrum;
(2) Since they are viruses, phages can be seen by the immune system of the host as a potential invader and may therefore rapidly be eliminated from the systemic circulation by reticulo-endothelial system clearance before they are accumulated in the target sites, or, they may be inactivated by the adaptive immune defense mechanisms (Dabrowska et al., 2005), which may lead to the treatment failure (Merril et al., 1996);
(3) Another concern of phage therapy is the potential ability of bacteriophages to transfer their DNA from a bacterial cell to another. This could be responsible for the transfer of pathogenicity determinants and virulence factors, leading to the development of a new microbe or even more resistant bacteria (Brabban et al., 2005; Maiques et al., 2007). Therefore, the use of phages that are unable to package extra host DNA or of phages that use the host DNA to synthesize its own DNA would be preferred (Gorski et al., 2009). In addition, under certain conditions, lytic phages would be transformed into lysogenic phages, making it possible to transfer their own virulence factors to the host bacteria (Brussow, 2007);
(4) Pharmacokinetic characteristics of phages are barely known. Optimal dose, route of administration, frequency, and duration of treatment still need to be defined before widespread clinical trials are contemplated;
(5) Phage therapy is time-sensitive. Use phages early in a disease setting could obtain a better therapeutic effect. For example, when the phages were given immediately after the infection of *E. coli* O18ac:K1:H7 ColV+, the efficacy was 100%; whereas when phage treatment was carried out 16 h after infection, the therapy failed (Smith and Huggins, 1982);
(6) Phages can cause the release of toxins, e.g., endotoxin (LPS), in large quantities from bacteria, especially Gram-negative bacteria. This may account for several side effects on the host such as the development of an inflammatory cascade leading to a multiple organ failure;
(7) Bacteria can obtain resistance to phages by mutation. The mutation rates for antibiotics and phages are 10^-^^7^ and 10^-^^6^, respectively (Carlton, 1999). There are at least four mechanisms that may be involved in bacterial resistance to a specific phage. Loss or lack of receptor (Liu et al., 2002), structural modification (Riede and Eschbach, 1986) and/or masking of the receptor (Drulis-Kawa et al., 2012) may prevent phage adsorption to the bacteria and prevent further an ability to generate new phages. The other mechanisms include the prevention of phage DNA integration by superinfection exclusion system (Sie), the degradation of phage DNA by restriction–modification defense system or by clustered regularly interspaced short palindromic repeats (CRISPR), and the blockage of phage replication, transcription, translation, or virion assembly by abortive infection system (Drulis-Kawa et al., 2012);
(8) Some phages can only survive in the intestines when bacteria counts reach certain numbers. Phages can only reduce but not completely eliminate *S. typhimurium* in the animal intestines (Berchieri et al., 1991; Callaway et al., 2011);
(9) Preparation of phages should be at a low temperature (Burrowes et al., 2011), and this kind of biological products are not stable.

Currently, the main challenge for the promotion of phage preparations is the lack of data obtained from large-scale clinical trials, thus hindering the universal application of them. Regulatory loopholes remain another major hurdle. In addition to the inherent safety concern, neither the US-FDA nor the European Medicines Agency has an approval process in place that can easily accommodate the ever-changing combinations of phages that accompanies the need to continuously develop the product in order to stay one step ahead of evolving MDR bacteria (Miedzybrodzki et al., 2012).

### ENDOLYSINS

Endolysins, including glucosidase, amidase, endopeptidase, and transglycosylase, are generated at the late phage lytic cycle, degrading bacterial peptidoglycan to facilitate the release of new phages from the infected bacteria. Endolysins were first discovered in the 1950s (Ralston et al., 1955), and revealed antibacterial activity against *Staphylococcus*,* Bacillus anthracis*, *L. monocytogenes*, and *Clostridium butyricum* in the 1990s (Low et al., 2005). Endolysins can treat sepsis and a few Gram-positive bacteria infections, such as *Enterococcus faecalis*, *C. perfringens*, and Group B *Streptococcus* (Fenton et al., 2010). Endolysin PAL is able to kill Group A *Streptococcus* which cause tonsillitis and other infections. Amidase PAL and endopeptidase Cpl-1 from phage Cpl-1 is capable of synergistically reducing the incidence of local and systemic pneumococcal disease (Loeffler et al., 2003; Fischetti, 2005). Endolysins LysK from phage K could kill nine *Staphylococcus*, including methicillin-resistant *S. aureus* (MRSA; O’Flaherty et al., 2005). Endolysins PlyV12 shows a good lytic activity against *Enterococci*, vancomycin-resistant *E. faecalis* and *E. faecium* (Yoong et al., 2004). Endolysins isolated from phage phi3626 can treat *Clostridium* infections (Courchesne et al., 2009).

Endolysins can quickly kill susceptible strains with wider antibacterial spectrum than phages and the activities are easier to be detected. Long-term evolution of endolysins makes them target only for some of the key elements on bacterial cell walls (Loeffler et al., 2003) thus cause bacteria lysed fast. Therefore, there is not enough time for bacteria to develop a resistance (Fischetti, 2005). However, the conventional methods for endolysin production are complicated, making the cost higher than that of the phage production. The use of recombinant DNA technology can reduce the costs (Loeffler et al., 2001), but it cannot guarantee the correct structure and full activity of the enzymes. Meanwhile, endolysins are easily degraded and lose activities during use and storage; hence they should be used with a full dose. In addition, the efficacy of endolysins is poor against Gram-negative bacteria; thus the use of single type of endolysin limits the scope of application. Future studies will focus on the chimeric endolysin (O’Flaherty et al., 2009), which could widen the antibacterial spectrum of this kind of enzymes. Presently, there are no published clinical studies concerning the endolysins.

### VIRION-ASSOCIATED PEPTIDOGLYCAN HYDROLASES

Bacteriophage virion-associated peptidoglycan hydrolases (VAPGHs) are a kind of phage lyases that hydrolyze bacterial peptidoglycan to assist the entry of phages into the bacterial cells (Rodriguez-Rubio et al., 2013). Many VAPGHs have been discovered and proved exhibiting antibacterial activities. Protein HydH5 from phage phiIPLA88 shows higher activity against *S. aureus* in the early logarithmic growth phase (Rodriguez et al., 2011). Protein 17 from phage P68 and protein gp61 from phage phiMR11 exhibit lytic activity against *S. aureus* including MRSA (Takac and Blasi, 2005; Rashel et al., 2008). Protein P5 from phage Φ6 possess the antibacterial activity against *Pseudomonas*, *E. coli*, *S. typhimurium*, *Proteus vulgaris*, and other Gram-negative bacteria whose outer membrane structures are unstable (Caldentey and Bamford, 1992). Protein Gp181 from phage ΦKZ demonstrates a lytic activity against the above mentioned Gram-negative bacteria as well as *Ralstonia solanacearum* and *Yersinia*. Protein gp36 of phage ΦKMV is effective against *P. aeruginosa* and *E. coli*, and exhibits a high thermal stability as the enzyme retained 26% of activity after incubation at 100°C for 2 h (Lavigne et al., 2004).

Currently, the researches on purified VAPGHs are limited. Since bacterial resistance caused by endolysin has not been reported to date, this may also be the case for VAPGHs (Rodriguez-Rubio et al., 2013). VAPGHs from Gram-negative bacterial phages have broad antimicrobial spectrum while the antibacterial spectrum of VAPGHs from Gram-positive bacterial phages only confine to specific host bacteria. VAPGHs are also effective against some antibiotic-resistant pathogens, increasing their application prospects (Paul et al., 2001; Rodriguez-Rubio et al., 2012). Many VAPGHs exhibit thermal stability, retaining activity at high temperatures generally associated with food technology. Taking into account that extracellular action can reduce the intracellular action which may trigger widespread bacterial resistance mechanisms, such as efflux pumps, VAPGHs provides an alternative source for bacterial lyases except for endolysins (Rodriguez-Rubio et al., 2013).

## ANTIMICROBIAL PEPTIDES

Antimicrobial peptides are classified into two categories, non-ribosomally synthesized AMPs and ribosomally synthesized AMPs, according to the peptide synthesis mechanism. The non-ribosomal AMPs, mainly produced by bacteria, are synthesized by peptide synthetases and structural modifications. They include gramicidin, polymyxin, bacitracin, and sugar-peptide. Polymyxin is from *B. polymyxa*, playing bactericidal effect by destroying the bacterial cell membranes. It is effective against many Gram-negative bacteria, such as *P. aeruginosa*, *E. coli*, *Klebsiella pneumoniae*, *Haemophilus*, and *Salmonella*. Bacitracin is a cyclic peptide produced by *B. subtilis* and *B. licheniformis*, and it functions by inhibiting the synthesis of cell wall peptidoglycans and glycoprotein core oligosaccharides in Gram-positive bacteria. Bacitracin shows bactericidal effects against Gram-positive bacteria, Gram-negative cocci and spirochetes. The USA and China have approved the use of bacitracin zinc and bacitracin methylene salicylic acid as feed additives in 1960 and 1990, respectively. These two bacitracins have a wide compatibility with other medicines.

Ribosomally synthesized AMPs can be further classified according to the sources of the peptides, such as mammals, amphibians, insects, plants, bacteria, viruses, etc. These AMPs are not only anti-bacterial or anti-mycotic, but also anti-protozoal, anti-viral, or anti-neoplastic, with broad application prospects. The positive charges of AMPs could form electrostatic adsorption with negatively charged phospholipid molecules on the bacterial cell membranes, resulting in structural damage of the membranes.

There are many reports on the protective effect of AMPs on humans (Guani-Guerra et al., 2010) and animals (Leonard et al., 2012). Here, bacteriocins which are produced by bacteria are taken as an example. Bacteriocins are defined into four classes as lantibiotics, the small heat-stable peptides (SHSPs), the large heat-labile proteins (LHLPs), and undefined mixture proteins with lipids and carbohydrates (Bierbaum and Sahl, 2009). Bacteriocins can also be subdivided on the basis of their modifications into class I (modified) and class II (unmodified or circular; Cotter et al., 2013). There have been lots of identified bacteriocins such as nisin, lactacin, lactocin, helveticin, fermenticin, sakacin, lacticin, plantacin, subticin, etc. *In vitro* tests show that bacteriocins have strong killing and suppressive effects on a variety of pathogens, including resistant pathogens (Field et al., 2011). In 1988, nisin received the US-FDA approval as food additive for the first time. Pediocin PA-1 from *Pediococcus* is on the market now. However, pure bacteriocins have so far only few and limited authorized uses in foods.

Bacteriocins have been found to have many distinct mechanisms of action. In addition to punch holes in the cell membrane like other AMPs do (Cotter et al., 2005), nisin, several lantibiotics and some class II bacteriocins, target lipid II (Bierbaum and Sahl, 2009), a key intermediate in the peptidoglycan biosynthesis machinery. Besides, bacteriocins can kill bacterial cells by interfering with DNA, RNA, and protein metabolism. MccB17 functions by inhibiting DNA gyrase-mediated DNA supercoiling, thereby interfering with DNA replication (Parks et al., 2007). MccC7-C51 inhibits aspartyl-tRNA synthetase, thus blocking mRNA synthesis (Metlitskaya et al., 2006). Nocathiacins and several other thiopeptides target the bacterial ribosome, binding the 23S rRNA of the 50S ribosomal subunit (Bagley et al., 2005).

Although AMPs are with good bactericidal effects and easily digested by bodies without adverse effect to the taste of feed or polluting the environment, a number of constraints have accompanied with the deepening research concerning AMPs.

(1) The high production cost limits the use of AMPs as effective antibiotic alternatives to livestock. Nowadays, bacteriocins are produced traditionally by culturing the wild strains, but the yield is low and the purification process is complex;
(2) Scientists begin to use genetic engineering techniques to synthesize bacteriocins owing to their peptide nature because they are directly encoded by genes (Cotter et al., 2013). AMPs can be modified by protein engineering to improve their efficacy. Site-saturation mutagenesis approach is used to create a bank of nisin A derivatives to screen the ones exhibiting enhanced bioactivity (Molloy et al., 2013). However, it cannot guarantee that the spatial structures of AMPs obtained by genetic engineering are consistent with the those of natural AMPs, which often causes differences in activities (Field et al., 2011);
(3) Many natural AMPs, such as melittin (a peptide which is the principal active component of bee venom), buthotoxin (a scorpion venom polypeptide) and plant AMPs, are potentially toxic to eukaryotic cells due to their hemolytic effects. Recently, a potent *E. coli* displaying multimeric AMPs on the cell surface was constructed (Shin et al., 2013). The multimeric AMPs can be converted into active AMP monomers by the pepsin in the stomach of livestock;
(4) Antibacterial spectrum of most bacteriocins is narrow, only effective to the related bacterial species (Lee and Kim, 2011);
(5) Bacteria can still develop resistance to AMPs by reducing the corresponding receptors (Piper et al., 2009), changing the composition of cell wall (Kramer et al., 2006), or changing the primary target of bacteriocin (del Castillo et al., 2001). Some bacteria can also utilize immune genes (Draper et al., 2009) or bacteriocin-hydrolase (Sun et al., 2009; Nocek et al., 2012) to obtain resistance. In fact, the widely used polymyxin AMPs has caused serious resistance in clinically important bacterial species, in both human and veterinary medicine;
(6) *In vivo* studies about pharmacodynamics, pharmacokinetics, and stability of AMPs are few. It is unclear whether AMPs or their metabolites are harmful to the body, and the immune response and other issues still need to be verified;
(7) AMPs are unstable during transportation, and are easily hydrolyzed by proteases in the alimentary canal during use.

## PRO-, PRE-, AND SYNBIOTICS

### PROBIOTICS

Probiotics have been defined by the World Health Organization as “microorganisms which, administered live and in adequate amounts, confer a benefit to the health of the host.” Probiotics are considered to be able to destroy pathogenic microorganisms by producing antimicrobial compounds such as bacteriocins and organic acids, improve gastrointestinal microbial environment by adherence to intestinal mucosa thereby preventing attachment of pathogens and competing with pathogens for nutrients, stimulate the intestinal immune responses and improve the digestion and absorption of nutrients. The commonly used probiotics include *Bacillus*, *Lactobacillus*, *Lactococcus*, *Streptococcus*, *Enterococcus*, *Pediococcus*, *Bifidobacterium*, *Bacteroides*, *Pseudomonas*, yeast, *Aspergillus*, and *Trichoderma*, etc. Microbiological feed additives used in EU mainly include *Bacillus* (*B. cereus* var. *toyoi*, *B. licheniformis*, *B. subtilis*), *Enterococcus* (*E. faecium*),* Lactobacillus* (*L. acidophilus*, *L. casei*, *L. farciminis*, *L. plantarum*, *L. rhamnosus*), *Pediococcus* (*P. acidilactici*), *Streptococcus* (*S. infantarius*), and some fungi such as *Saccharomyces cerevisiae* and kluyveromyces (Anadon et al., 2006). Japan started using probiotics in 1960s, and China began the application of probiotics in 1980s. US-FDA approved 42 probiotics till 1989 (Gaggia et al., 2010). In 2000, the total sale of feed probiotics worldwide was $186 million.

However, the probiotic market is very less organized and the supervision and management of probiotic production are defective. For instance, China Ministry of Agriculture has approved 12 probiotics, but there are more than 50 probiotics being used in China. Due to lack of standards, animal poisoning, allergies, and diarrhea after using probiotics are reported from time to time. Despite years of experiences in the use of lactobacilli and bifidobacteria which are proved to be safe, the safety of other species still need to be examined (Borriello et al., 2003). Meanwhile, probiotics are potentially harmful to the congenitally immunodeficient animals (Balish and Wagner, 1998). Besides, previous studies have failed to consistently prove the beneficial effects of probiotics in animals. For example, microcin-producing *E. coli* could inhibit the growth of *Salmonella in vitro*, but the results *in vivo* were not satisfactory (Frana et al., 2004). Contradictory results with very heterogeneous studies are reported on the treatment and the prophylaxis of upper respiratory tract infections (Alexandre et al., 2014). Furthermore, probiotics have adverse effect on the normal gut flora. *Lactobacillus* and *Bacillus* can destroy the ecological balance of normal flora in the body, which may be related to the occurrence of urinary tract infections and other diseases. Dietary cider yeast can potentially alter the gut microbiota. However, such changes depend on their endogenous microbiota that causes a divergence in relative response to that given diet (Upadrasta et al., 2013).

For putting probiotics into practice as feed additives, the followings are challenged: (1) the number of safe bacterial species is limited; (2) microbial preparations are easily inactivated in feed processing, transport, and storage processes; (3) they cannot withstand low pH in gastrointestinal tract and bile acids during use; (4) it is difficult to reach high enough number of viable cells to colonize in the intestine. In addition, because there are no adequate corresponding regulations and standards, probiotic products cannot be labeled with proper dose, indicated in suitable animal target as well as other factors that may affect the efficacy.

### PREBIOTICS

Prebiotics are non-digestible (by the host) food ingredients that have a beneficial effect through their selective metabolism in the intestinal tract (Gibson et al., 2004). Prebiotics include oligosaccharides [such as fructooligosaccharide (FOS), mannan-oligosaccharide (MOS)], polysaccharides, natural plant extracts, protein hydrolysates, polyols, etc. Prebiotics can selectively proliferate intestinal bacteria, promote immune functions and show anti-viral activity. Some of them are able to promote mineral absorption and regulate metabolism. The applications of prebiotics as feed additives began in the late 1980s. China began to use them in the late 1990s. Currently, the most promising prebiotics are multifunctional oligosaccharides and acidifiers.

Prebiotics are stable compounds with no residue, no induced resistance, and wide variety of sources. However, nowadays in EU many prebiotic products are not authorized as feed additives under the commission regulation (EC) 1831/2003 (European Commission, 2003). This is due to some drawbacks of this kind of products. First, prebiotics themselves cannot inhibit and kill pathogens, thus they cannot prevent or treat bacterial infections as antibiotics do. Second, feeding with large quantity of prebiotics may cause bloating, diarrhea, and other adverse reactions due to the fermentation in the gastrointestinal tract (de Vrese and Schrezenmeir, 2008). Third, a study suggests that the prebiotic role of mannose is related to its oligosaccharide structure (Badia et al., 2013). Both β-galactomannan and MOS from yeast *Saccharomyces cerevisiae* attenuates *Salmonella*-induced secretion of IL6 and CXCL8, but cells treated with monosaccharide D-mannose show similar levels of these proinflammatory factors compared with the control of infection. Since the relationship between structure and physiological function of prebiotics is not clear, the efficacy of prebiotics is always variable with different animal species, ages, and physical conditions. Sometimes, mutual antagonism occurs. In addition, the high cost of prebiotic production limits their application in the animal husbandry industry.

### SYNBIOTICS

Synbiotics are the joint preparations of probiotics and prebiotics, and thus have the dual role of them (Andersson et al., 2001). There are some reports on the effect of synbiotics on the physiological and biochemical indexes of piglets including the enhancement of immune function in piglets, the improvement of average daily gain and digestibility, the reduction of diarrhea morbidity and mortality, the ease of weaning stress response, and the significant promotion of piglet performance (Gaggia et al., 2010). However, the reports of the beneficial effects of synbiotics on swine production are still limited (Modesto et al., 2009). The mixing proportions of probiotics/prebiotics for the majority of synbiotics are inadequate (Kolida and Gibson, 2011), thus resulting in a non-synergistic effect. So far, synergy mechanism of probiotics and prebiotics has not been thoroughly understood; hence, the extensive application of synbiotics has a long way to go.

## PLANT EXTRACTS

Plant materials are used widely in traditional systems of medicine (Savoia, 2012). Plant extracts, also known as phytobiotics, have been exploited in animal nutrition, particularly for their antimicrobial, anti-inflammatory, anti-oxidative, and anti-parasitic activities (Vondruskova et al., 2010; Hashemi and Davoodi, 2011). Many plants have beneficial multifunctional properties derived from their specific bioactive components. Biologically active constituents of plants are mostly secondary metabolites, such as terpenoids (mono- and sesquiterpenes, steroids, etc.), phenolics (tannins), glycosides, and alkaloids (present as alcohols, aldehydes, ketones, esters, ethers, lactones, etc.; Huyghebaert et al., 2011). Among 109 new antibacterial drugs, approved in the period of 1981~2006, 69% originated from natural products, and 21% of the antifungal drugs were natural derivatives or compounds mimicking natural products (Newman, 2008).

Plant extracts are generally considered safe and effective against certain bacteria. They are extensively used in feed as growth promoters and health protectants (Hashemi and Davoodi, 2011; Abreu et al., 2012), particularly in Asian, African, and South American countries, and are gradually used in developed countries in recent years. In pig production, it is thought that oregano, cinnamon, Mexican pepper, thyme, oregano, and *Camellia sinensis* can decrease pathogenic microbial mass in the intestines (Manzanilla et al., 2004; Namkung et al., 2004; Zanchi et al., 2008); sangrovit, aged garlic extract, and allicin are able to increase body weight gain (Borovan, 2004; Tatara et al., 2008); thyme, clove, oregano, eugenol, and carvacrol are capable of improving pig performance (Oetting et al., 2006; Costa et al., 2007). Effects of phytogenic feed additives on the production performance of poultry are also reported (Hashemi and Davoodi, 2010).

It is considered that plant extracts at minimum inhibitory concentrations (MICs) of 100~1000 μg/ml in the *in vitro* bacterial susceptibility tests possess antibacterial activities (Simoes et al., 2009). Useful antimicrobial phytochemicals can be divided into several categories, such as phenolics/polyphenols, terpenoids/essential oils, alkaloids, lectins/polypeptides (Windisch et al., 2008). Phytochemicals exert their antimicrobial activity through different mechanisms. For example, (1) tannins act by iron deprivation and interactions with vital proteins such as enzymes (Scalbert, 1991); (2) the main indoloquinoline alkaloid, cryptolepine, is a DNA intercalator and an inhibitor of topoisomerase (Karou et al., 2006); and (3) saponins form complexes with sterols presenting in the membrane of microorganisms, causing membrane damages and consequent collapse of cells (Morrissey and Osbourn, 1999). Essential oils have long been recognized for their antimicrobial properties (Lee et al., 2004), but the exact antimicrobial mechanism is poorly understood. In fact, the antimicrobial activities of many plant extracts have not been elucidated clearly yet (Stavri et al., 2007). Some *in vivo* observations support the assumption that the general antimicrobial potential of phytogenic feed additives is contributed to a final reduction of intestinal pathogen pressure (Windisch et al., 2008). To the year of 2008, only two kinds of human-used antibacterial plant extracts have completed the clinical trials, and additional 13 plant extracts are under clinical trials (Harvey, 2008).

A common feature of phytobiotics is that they are a very complex blend of bioactive components. There is a lot of variations in the composition of phytobiotics due to the biological factors (plant species, growing location, and harvest conditions), manufacturing (extraction/distillation and stabilization) and the storage conditions (light, temperature, oxygen tension, and time; Huyghebaert et al., 2011). Only under certain circumstances, plant extracts could improve animal performance and control diseases. Bird growth responses to herbal plants are still controversial, since the exact quality as well as the quantity of the active chemicals in plant extract are required to determine the response for bird performance (Cross et al., 2007), which are often lacking. Some parameters that affect the efficacy of the phytobiotics mainly include the plant parts and their physical properties, the genetic variation of the plant, age of the plant, different dosage used, extraction method, harvest time, and compatibility with other ingredients (Yang et al., 2009). In addition, the beneficial effect of dietary phytobiotics can be influenced by the nutritional status of animals, the infection, the diet composition and the environment condition (Giannenas et al., 2003).

It is difficult to perform systematic and comprehensive toxicology studies and safety assessment on herbs and their extracts, due to their complex composition. The challenge is to identify and quantify the multitude of actions in order to claim improving feed utilization, animal physiology and health status. Currently, herbal feed additives on the market do not meet the “trace and efficient” principle for feed additives. They are commonly used at very large dose, generally at feed ratio of 1–2%, some up to 5%, and this may affect the nutrition of a feed. Another consideration when using phytogenic feed additives is the possible interactions with other feed additives. There are reports on the adverse interactions of phytogenics with enzyme preparations (Sarica et al., 2005) and with protein through partial denaturation (Anadon et al., 2005). In summary, although phytobiotics are a group of natural additives, researches on their mechanisms of action, compatibility with diet, toxicity and safety assessment need to be done before they can be applied more extensively in animal feeds.

## INHIBITORS TARGETING PATHOGENICITY

### QUORUM SENSING INHIBITORS

Bacterial pathogenicity is, in part, under the regulation and control of QS system (Swift et al., 2001). QS system consists of self-induced signaling molecules (autoinducers, AIs), receptors, and downstream regulatory proteins. AIs are *N*-acyl homoserine lactones (AHLs) secreted by Gram-negative bacteria, autoinducing peptide (AIPs) secreted by Gram-positive bacteria, autoinducer-2 (AI-2), and other signaling molecules such as quinolones, esters, and fatty acids.

Inhibitors targeting QS can block the functions of QS system and therefore prevent bacterial virulence regulated by QS system. QS inhibitors (QSIs) are classified into three groups including non-peptide small molecule, peptide (mainly AIPs homologs), and protein QSIs. Non-peptide QSIs mainly include AHLs analogs, such as ACP homologs, L/D-*S*-adenosylhomocysteine and butyryl-*S*-adenosyl-L-methionine (Parsek et al., 1999), which can interfere with the synthesis of QS signal molecules or the binding to the receptors. Mice treated with synthetic AIP-II had resistance to *S. aureus* infection (Mayville et al., 1999) and treated with furanone observed the decrease of virulence of *P. aeruginosa* (Hentzer et al., 2003). QS quenching enzymes and QS quenching antibodies are proteinaceous QSIs (Amara et al., 2011). The former, such as AHL-acylase, lactonase, oxidoreductases from *Rhodococcus* and paraoxonase from mammals, degrade signaling molecules. Human and murine paraoxonases 1 show the host modulators of *P. aeruginosa* QS (Ozer et al., 2005). In addition, competitive organisms are able to clear the signal molecule to quench QS (Kalia and Purohit, 2011). For instance, *E. coli* ingest AI-2s to influence the QS of *Vibrio harveyi* (Xavier and Bassler, 2005). Bacteria with AHL-degrading activity protect *Artemia* spp., rotifers and larvae of turbot or prawn from infection (Nhan et al., 2010). In animal serum, apolipoprotein B (ApoB) bind with AIP1 molecules of *S. aureus*, effectively reducing its QS (Peterson et al., 2008).

Importance of QSIs is inferred from a growing number of patents related to this field in the last few years and extensive researches in this field (Kalia and Purohit, 2011; Romero et al., 2012). QSI appears to be effective *in vitro* and in various animal models; however, all the structural classes of compounds that have been studied and patented have limitations when used *in vivo* (Bhardwaj et al., 2013):

(1) Except for LED209 that has entered pre-clinical trial, the vast majority of QSIs cannot be widely applied because of their toxicity to eukaryotic cells. Penicillic acid and patulin are toxic to human cells, and halogenated furanone has carcinogenic toxicity (Bjarnsholt and Givskov, 2007);
(2) Although QSI itself does not interfere with the growth of bacteria and thus dose not cause selective pressure on bacteria, it is still possible for bacteria to develop new resistance (Kalia and Purohit, 2011). For example, mutations of bacterial AHL synthases and QS signal receptors (e.g., LuxR) remain (Kalia and Purohit, 2011);
(3) Drugs based on AHL analogs could suffer from hydrolysis of lactone ring and drugs based on proteins could have stability problems. For example, lactonases produced in the human airway epithelia show to quench QS signals from *P. aeruginosa* (Chun et al., 2004). Therefore, special caution needs to be exercised in the development of synthetic anti-QS analogs based on the lactone core (Sintim et al., 2010). Unraveling the structures of molecules like AI-3 and deciphering the structural nuances of AI-2 receptors, LuxS and the myriad signal transduction cascades involved in QS processes are of utmost importance;
(4) Bacterium only use its specific signal molecules; therefore, it is difficult to find a broad-spectrum QSIs (Bjarnsholt and Givskov, 2008). Broad-spectrum QS quenching enzymes have good prospects, but the possibility that protein QSIs may cause host immune response should be considered;
(5) The degradation of QS signaling molecules will affect the normal activities of the host intestinal flora (Amara et al., 2011);
(6) Expression of virulence factors in biofilm-forming bacteria is much lower than that in the planktonic bacteria (Resch et al., 2005); hence, one has to consider the actual value to use QSIs for treating pathogens which have generated biofilms.

It is reported that bacteria are more sensitive to the antibiotics when the antibiotics are used in combination with QSIs. Therefore, combination usage serves a better strategy to enhance the antimicrobial effects and prevent the bacterial resistance.

### BIOFILM INHIBITORS

Biofilms are structured consortium of bacteria embedded in a self-produced polymer matrix consisting of polysaccharide, protein and DNA. Biofilm-forming bacteria may cause chronic infections because they show increased tolerance to antibiotics and disinfectant chemicals as well as resisting phagocytosis and other components of the body’s defense system (Hoiby et al., 2010). As for treating staphylococcal biofilm, protein synthesis inhibitors (e.g., oxazolidinones and tetracyclines), cell membrane and wall-active antibiotics (e.g., lipopeptides and glycopeptides) and inhibitors for DNA and RNA synthesis (e.g., rifampin) are often used (Kiedrowski and Horswill, 2011). Methane-thiosulfonate and mercurial *p*-hydroxymercuribenzoic acid could target sortases, a membrane enzyme catalyzing the covalent anchoring of surface proteins to peptidoglycans, which are involved in bacteria adhesion (Chen and Wen, 2011).

Biofilm formation involves bacterial cell adhesion, QS regulation, biofilm maturation, and bacteria spread; therefore, a single drug is difficult to completely remove pathogens in biofilms. Combination therapy is a wise choice, which generally uses antibiotics with antibiotics or antibiotics with biofilm inhibitor as the combination. Combination of both fluoroquinolones and macrolides or fosfomycin seems to be most effective regimen against biofilm infections in urinary tract (Kumon, 2000). Another promising strategy is the use of enzymes that can dissolve the biofilm matrix [e.g., polysaccharide hydrolases (Kaplan et al., 2004), DNases (Izano et al., 2008), proteases (Marti et al., 2010), and alginate lyases] as well as quorum-sensing inhibitors that increase biofilm susceptibility to antibiotics. It is shown that administration of DNase and alginate lyase enhances the activity of tobramycin against biofilms by dissolving the biofilm matrix of *Pseudomonas aeruginosa* (Alipour et al., 2009). In addition, monoclonal antibody of alginate shows certain damaging effects against *P. aeruginosa* biofilms (Mai et al., 1993), and enhances the ability of antibiotic to penetrate biofilms (Hatch and Schiller, 1998). Urokinase or lumbrokinase combining with fleroxacin significantly enhances the inhibition of *P. aeruginosa* biofilms (Selan et al., 1993). This combination not only degrades the polysaccharide protein complex in the bacterial biofilms, but also affects the bacterial DNA synthesis. Efflux pump inhibitor in combination with miconazole can effectively remove *Candida albicans* from the biofilm (Qi and Wang, 2009).

The way from molecular mechanisms of biofilm formation to anti-biofilm products is promising, but still a long one. Although biofilm inhibitors can inhibit biofilm formation, they do not inhibit bacterial growth or kill bacteria. Hence, when biofilm inhibitor use is discontinued, bacteria will produce biofilm again to protect themselves against the adverse environmental conditions.

### BACTERIAL VIRULENCE INHIBITORS

An important emerging strategy to combat bacteria seeks to block the ability of bacteria to harm the host by inhibiting bacterial virulence factors. Development of compounds inhibiting the function and transmission of bacterial toxins is a novel anti-infective strategy. The protein complex of anthrax toxin contains lethal factor (LF), edema factor (EF), PA, and other components. Single component is non-toxic, but the combination of LF or EF with PA will lead to a pathological effect (Young and Collier, 2007). A small molecular, hydroxamate (LFI), can bind to the active site of LF, inhibiting the activation of LF and preventing anthrax infection (Shoop et al., 2005). Cisplatin shows inhibitory effect to PA heptamer assembly, thus blocks the toxicity of LF and EF. However, only simultaneous feeding of cisplatin and a lethal amount of anthrax toxin has a protective effect on rodents, while delayed feeding of cisplatin would have resulted in a failure (Moayeri et al., 2006). Cholestyramine can bind with clostridial toxin to prevent its adsorption to intestinal epithelial cells, thus weakening the toxicity cause by the toxin.

The type three secretion system (T3SS) is found in over two dozens of Gram-negative bacteria and functions by injecting effector proteins directly into the cytosol of the host cells. One acyl salicylaldehyde compound targeting the T3SS of *Yersinia pseudotuberculosis* prevents the translocation of effector molecules, and thus attenuates the pathogens (Nordfelth et al., 2005). Different acyl salicylaldehydes inhibit two T3SS effector molecules to prevent *Salmonella* invasion of the intestinal epithelial cells (Hudson et al., 2007). Currently, studies on the acyl salicylaldehyde under bovine intestinal model are still limited.

Prevention of bacterial adhesion is equally important. Pilicides inhibit bacterial pili formation. Pyridone and its N-terminal amino acid derivatives competitively inhibit the chaperone protein binding to pilus protein. Pyridone is also able to inhibit blood clotting and biofilm formation, thus inhibiting bacterial adhesion (Pinkner et al., 2006). Regulating the expression of bacterial virulence factor is meaningful as well. For instance, virstatin inhibits the expression of *Vibrio cholerae* toxin and pilus, thereby prohibiting the colonization of *V. cholerae* in the gut (Hung et al., 2005). Virulence regulation can be achieved through different aspects, such as interfering bacterial QS (Clatworthy et al., 2007). Inhibition of the downstream effects of toxin is feasible. For example, Cl^-^ secretion inhibitors can moderate diarrhea.

Toxin inhibitors carry out the function by disrupting the bacterial toxins, or modulate the host responses to the toxins. They do not directly inhibit bacteria, thus causing no bacteria resistance selection pressure. However, like the biofilm inhibitors, even in the presence of toxin inhibitors, bacteria can still grow and reproduce. Therefore, in the absence of these substances, bacteria may produce toxins and exhibit virulence again.

## FEED ENZYMES

The nutrients for the multiplication and growth of bacteria in the intestinal tract are derived largely from dietary components, which are either not digested by digestive enzymes or absorbed so slowly that bacteria in host guts compete for them. Exogenous enzymes not only influence the absorption of nutrients but also produce nutrients for specific populations of bacteria through their action (Bedford and Cowieson, 2012). Therefore, their use has a direct impact on the microfloral populations (Apajalahti et al., 2004).

The most widely used feed enzymes are mixture of a variety of glycanases, and the single-using degrading enzyme is phytase (Ravindran and Son, 2011). Recombinant synthesized enzymes such as phytases and carbohydrases are commercially produced and sold as feed additives in monogastric food-animal production (Adeola and Cowieson, 2011). Phytase has significant effects on the digestibility of calcium, phosphorus, and minerals as well as the intestinal mucin production and the endogenous losses, all of which influence the nutrient supply and the intestinal environment which will alter the selection pressures on bacterial species (Bedford and Cowieson, 2012). Xylanase added to a wheat-based diet alleviates the pathological effects of *C. perfringens* in broiler chickens (Liu et al., 2012a). Dietary use of encapsulated lysozyme, as a feed additive in the diet of chickens significantly reduced the concentration of *C. perfringens* and gastrointestinal lesions due to the organism in the ilium (Liu et al., 2012b).

Despite the mature phytase market, other kinds of feed enzymes have different defects. Generally, the activity of the enzyme is low; the cost is high; and the production and quality control lack standards. The stability and activity of feed enzymes depend mainly on the production process (Slominski, 2011). It is well recognized that animal responses to feed enzyme additives are not entirely predictable, and these inconsistencies could be attributed *inter alia* to the enzyme type, the amount of enzyme applied, the presence of enzyme side activities, the diet composition and the animal variation (Ravindran and Son, 2011). The mechanisms by which feed enzymes influence the intestinal microbiota have been known for some time, e.g., through enhancing nutrient delivery to the host and by provision of fermentable oligosaccharides. It is still unknown, however, to which extent this effect contributes to the net benefit of the enzyme use, nor is it clear which are the major microbial species involved (Bedford and Cowieson, 2012). Recent work shows that if the use of enzymes is to replace prophylactic antibiotics, then the target must be to enrich the ileum and cecum with *Clostridium* cluster XIVa species and *E. coli* while depressing the numbers of *Lactobacillus* spp. (Smulikowska et al., 2010). Until an accurate description of a desirable flora can be documented, the feed enzymes can be appropriately used.

Since enzymes do not directly attack bacteria, but only reduce the substrates for the growth of bacteria, the antibacterial effect is not obvious. Glucose oxidase (GOD), mainly from *Aspergillus niger* and *Penicillium*, can specifically oxidize D-glucose to produce gluconic acid and hydrogen peroxide (Geisen, 1999). When hydrogen peroxide accumulates to a certain level, it can be harmful to intestinal bacteria and inhibits their propagation while consuming oxygen in the intestine, which may help the growth of *Bifidobacterium* and *Lactobacillus* and other beneficial anaerobic bacteria. An enzyme alginogel composed of GOD, lactoperoxidase, and guaiacol was able to prevent the formation of biofilms and inhibit the established biofilms (Cooper, 2013). Given that the mechanism of action of this enzyme is different from other feed enzymes, the related feeding effects of GOD are worthy of further study.

## PERSPECTIVES

Ideal alternatives to antibiotics should: (i) have non-toxic or no side effects on animals, (ii) be easy to eliminate from the body or consist of short term of residues, (iii) not induce bacterial resistance, (iv) be stable in the feed and animal gastrointestinal tract, (v) be easily decomposed and not affect the environment, (vi) not affect palatability, (vii) not destroy the normal intestinal flora of animals, (viii) kill or inhibit the growth of pathogenic bacteria, (ix) enhance the body resistance to the disease, (x) improve feed efficiency and promote animal growth, and (xi) have good compatibility. In fact, there are no alternatives to antibiotic that currently meet all the above mentioned requirements.

There is still a considerable gap between antibiotic alternatives and antibiotics concerning the effectiveness of disease prevention and growth promotion. Antibacterial vaccines are generally used for the prevention of bacterial infections, and currently only a small number of bacterial infective diseases can be controlled by vaccines. Immunomodulators and feed enzymes mainly preserve the health of animals, but do not directly kill or inhibit bacteria. Bacteriophages are currently only used in food, and the safety is still questionable. The composition of plant extracts and probiotics is complex and the quality in terms of stability is poor, resulting in varying effects and safety risks. Inhibitors targeting QS and virulence of bacteria are still in research with no approved products, and most QSIs are toxic to eukaryotic cells. Biofilm inhibitors show good results only when used in combination with antibiotics. Although AMPs can treat bacterial infections, the high cost and narrow antibacterial spectrum restrict their wide use, and they can still induce bacterial resistance. Meanwhile, proteinaceous compounds, for example, feed enzymes and AMPs that have been put into market as well as bacteriophage lysins, QS quenching enzymes and enzymatic biofilm inhibitors under development, are naturally unstable and easily degraded in the digestive tract. On the other hand, antibiotics can directly inhibit or kill bacteria with better antibacterial effect than all antibiotic alternatives. Moreover, antibiotics are made by single and relatively pure active ingredient with high stability, consistency, and quality ensured by good manufacturing practice. Considering clinical efficacy, humans have not yet found a more effective way than selecting appropriate antibiotics for the treatment of indicated bacteria.

Antimicrobial resistance is the major reason for EU to ban low dose of antibiotics as feed additive. In fact, the penicillin’s producer, *Penicillium*, has coexisted with other bacteria for tens of thousands of years. Only after the extensive use of penicillin was the emergence penicillin-resistance discovered. Drug pressure is proportional to the chance of development and dissemination of drug resistance; therefore, although antibiotic alternatives have not been associated with bacterial resistance yet, with a large number of these alternatives in use, bacteria might mutate eventually to develop resistance. Like the precedent use of antibiotics, the irregular and illegal use of antibiotic alternatives may also bring negative issues.

Meanwhile, we must not forget that “it’s better to prevent than cure” (Bourlioux, 2013). For many developing countries, because of the poor farming environment and the high incidence of disease, antibiotics are still an effective tool in the prevention and control of animal diseases. It is shown that the ban of growth promoters in EU demands the improvement of the farm hygiene (Castanon, 2007). When the amount of “old” antibiotics used in feed reduces due to the ban, prevalence of bacterial infections in the target animals would likely increase without fundamental improvement of production environment. This may lead to the increase of therapeutic uses of advanced antibiotics and result in some unintended consequence that may cause new challenges for public health concern. Additionally, there are no scientific evidences to clearly separate the causal relationship between treatment use and prevention use of the antibiotics with respect to resistance development. The ultimate benefit and risk should be assessed before implementing such a policy as a result of political/social pressure, as bacteria may not necessarily “listen” to the policy-makers. Thus, the decision concerning the use of in-feed antibiotics should be made based on scientific approaches. The ban of antibiotics as growth promoters cannot be copied in every country of the world.

The efficacy of traditional antibiotics can still be improved. Some “old” antibiotics can find new bacterial targets and reinforce the anti-infectious therapy toward some MDR bacteria. It has been demonstrated that in many cases, there are non-carbapenem alternatives for the treatment of extended-spectrum-β-lactamase-producing *E. coli* (ESBL-Ec) infections (Fournier et al., 2013). Besides, new formulations can allow targeted drug delivery via nanoparticles and the association of molecules can reinforce the antimicrobial effect of antibiotics (Bourlioux, 2013). Furthermore, in empirical therapy, use of broad-spectrum bactericidal agents that will eradicate the presumed infective microorganism(s), which potentially could be MDR, should be preferred. Once an infection is under control and the culture and susceptibility results are reported, it is important to switch to the most suitable narrow-spectrum agent thus decreasing the potential of adverse drug effects and the risk of development of antibiotic-induced resistance (Lynch, 2012).

In summary, reasonable use of antibiotics and continuous development of alternatives to antibiotics are needed to ensure the long-term sustainable development of animal husbandry. We must strictly define the target animals, duration of the treatment and the withdrawal period, for prudent use of antibiotics as well as regulation/policy making regarding their use. At the same time, we must strengthen the supervision and enforcement of laws in order to control antibiotic resistance and residues from the food chain within established safe levels. Furthermore, we must improve the management of animal nutrition and production hygiene, since recent European developments showed a distinctly more positive outcome of the ban of antibiotic growth promoters than was anticipated during the first years after the ban due to the improvement of animal welfare (Cogliani et al., 2011). The research of antibiotics alternatives will be a long process. In addition to research and development of new efficient and safe alternatives, we should strengthen the study concerning the effects of combined use of antibiotics and their alternatives aimed at maintaining a healthy agricultural economy and preservation of potent antibiotics for efficacious therapy in humans.

## Conflict of Interest Statement

The authors declare that the research was conducted in the absence of any commercial or financial relationships that could be construed as a potential conflict of interest.
